# WHAT DO 'BEHAVIORS" COST? UNDERSTANDING COSTS IN DEMENTIA CARE PARTNER-BASED RESEARCH

**DOI:** 10.1093/geroni/igac059.1549

**Published:** 2022-12-20

**Authors:** Walter Dawson, Sarah Gothard, Nora Mattek, Jeffrey Kaye, Allison Lindauer

**Affiliations:** Oregon Health & Science University, Portland, Oregon, United States; Oregon Health & Science University, Portland, Oregon, United States; Oregon Health and Science University, Portland, Oregon, United States; Oregon Health & Science University, Portland, Oregon, United States; Oregon Health & Science University, Portland, Oregon, United States

## Abstract

The out-of-pocket costs to care for individuals living with dementia are high, and is exacerbated by the expense of managing behavioral and psychological symptoms of dementia (BPSD) (e.g., depression, irritability). STELLA (Support via TEchnology: Living and Learning with Advancing AD) is a telehealth-based intervention that provides a personalized approach to teach care partners (CPs) strategies to manage BPSD. To understand the relationship between BPSDs and costs, weekly surveys were administered to CPs (n=12). Surveys asked about out-of-pocket costs incurred from: hospitalizations and emergency department (ED) utilization, primary care visits, use of paid in-home care, prescription drugs, and over-the-counter medications. The most frequent cost reported by CPs was prescription drug related (11 CPs), while costs associated with hospitalizations and ED were the least frequently reported (4 CPs). A longitudinal weekly survey-based approach to quantify CP costs is a novel approach examine an intervention outcome (cost) that matters to families and policymakers.

